# Assessment of the relative contribution of volume and concentration changes in Yttrium-90 labelled resin microspheres on ionization chamber measurements

**DOI:** 10.1007/s13246-017-0601-z

**Published:** 2017-11-17

**Authors:** Nicholas Forwood, Kathy P. Willowson, Michael Tapner, Dale L. Bailey

**Affiliations:** 1 Department of Nuclear Medicine, Royal North Shore Hospital, St Leonards, NSW Australia; 2 Faculty of Health Sciences, University of Sydney, Sydney, NSW Australia; 3 Institute of Medical Physics, University of Sydney, Sydney, NSW Australia; 4 Sirtex Medical Limited, Sydney, NSW Australia; 5 ABX-CRO, Dresden, Germany

## Introduction

Re-entrant ionization chambers or, “dose calibrators”, are the standard equipment used to assay radiopharmaceuticals in clinical practice. These devices consist of a gas-filled (usually argon), pressurized ionization chamber surrounding a well in which the source is placed. When calibrated they can provide a measurement of radioactivity with a linear response over a very wide range of values [1]. The readout of the device is the current recorded from the chamber multiplied by a calibration factor [2]. This calibration factor is specific for the isotope being assayed and the geometry of the source. Generally radioactivity in a glass ampoule or vial is used to measure the accuracy of the dose calibrator against a national standard. In the clinical setting the radioactivity can be measured using a variety of vessels and source volumes such as syringes and glass or plastic vials.

Most radionuclides used in nuclear medicine have a characteristic gamma emission so that the current being measured is predominantly caused by photons ionizing in the chamber without interacting within the source itself. For this reason, the size and shape of the source will have less of an impact on the activity reading of the calibrator for a gamma emitter than for an isotope with no characteristic gamma emission. Controlled experiments have shown that for ^99m^Tc the difference in activity reading is less than 5% when the volume of the source changes from ~ 0.5 ml in syringe to 9 ml in syringe [3]. Such a discrepancy is very significant in the science of metrology but in a clinical setting, where an accuracy of 10% is considered acceptable [4], it may not be an issue. Low energy gamma emitters are also sensitive to the geometry of the source and the response variation to source volume can be an order of magnitude higher for ^125^I than for ^99m^Tc [5]. Furthermore, the change from a glass ampoule to a plastic syringe affects the dose calibrator reading for ^125^I by 20% or more [6]. Variation in response from different syringes derived from the same batch have been found to be as high as 30% [6]. The equivalent test with ^90^Y was shown to lead to twice the variation in activity reading [3] which is clinically significant. In this case, the variation is because the reading in the dose calibrator comes not from the beta particles themselves but from the bremsstrahlung photons that are generated in the source and its container [7]. The spectrum of bremsstrahlung photons that are detected by the ionization chamber is highly dependent on the material in which it is contained and the medium through which it is attenuated.

^90^Y is a radionuclide of increasing importance in nuclear medicine due to its growing frequency of use in a variety of therapies. Whilst generally regarded as a pure beta emitter, it does produce a very small quantity (32 ppm) of positrons during the 0^+^→0^+^ de-excitation of ^90^Zr, to which ^90^Y decays, from pair production (β^+^–β^−^) and subsequent annihilation radiation [8, 9]. The lack of gamma emission has made this radionuclide difficult to calibrate accurately in the clinical setting with surveys indicating that less than half of commercial radionuclide calibrators were able to measure ^90^Y radioactivity in a vial to within an accuracy of 5% [10, 11]. The difficulties of calibrating solutions of ^90^Y have been studied and the results demonstrate that for ^90^Y the chamber response is highly dependent on the geometry in use and care should be taken to calculate the dial setting or calibration factor for each geometry and volume being used [12].

Resin-based ^90^Y labelled microspheres are now widely used in the treatment of primary and metastatic liver cancer. The microspheres are typically shipped in a glass vial and suspended in aqueous solution to a total volume of 5 ml. The amount of radioactivity can be measured in a dose calibrator both before and after a pre-determined volume of the suspension has been withdrawn for administration to the patient (Package Insert, SIR-Spheres microspheres, Sirtex Medical Limited, 2015; PE-IC-11, December 2013). The resin microspheres are denser than water and, after agitation, settle to the bottom of the vial. Measurement of radioactivity for ^90^Y-labelled microspheres will be affected by not only the volume to be dispensed for the treatment, the choice of vessel and the variability of the geometry of the vessel, but also the concentration of the microspheres in the sample and the concentration of the suspension. This work presents a novel method for comparing the relative sensitivity of an ionization chamber to changes in the volume and concentration of suspended ^90^Y resin microspheres measured in the glass vial provided by the manufacturer.

## Materials and methods

### Materials

Ten unused glass shipping vials used for dispensing of resin-based ^90^Y microspheres were obtained from Sirtex Medical (Sydney, Australia). Weights were measured using a precision balance (AdventurerPro AV53, OHaus Corporation, USA), calibrated within the previous 12 months by a certified authority. Radioactivity was measured in a CRC 55 TW dose calibrator (Capintec Inc, USA) using the “48 × 10” calibration setting which is recommended by the dose calibrator manufacturer for the measurement of ^90^Y. This calibration factor is specific for 5 g of solution in a standard source ampoule made of about 0.6 mm thick borosilicate glass. It is noted by the instrument manufacturer that this setting is for estimation purposes only.

### Weight measurement

The dry weight of each of the ten glass vials was measured using the balance without the rubber stopper and metal crimp that are normally part of the vial. Uncertainty in the measurement of weight was estimated by repeated measurement of a sample glass vial with 5 g water inside.

### Assay of normalized activity in solution

**Fig. 1:**
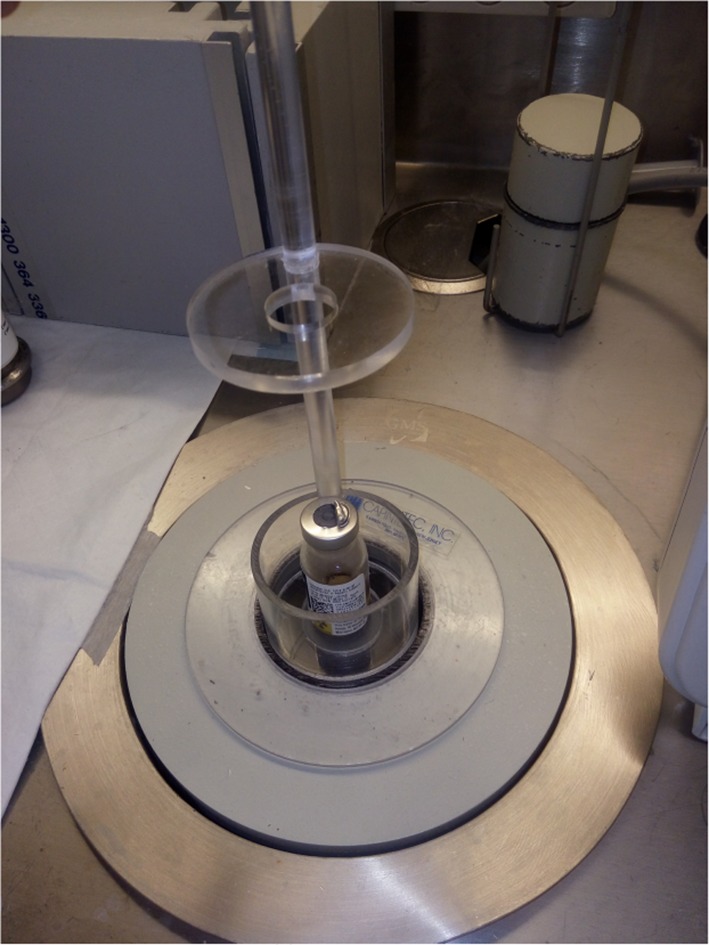
The source detector geometry for the assay of ^90^Y microspheres. The same measurement procedure was used to assay the ^90^Y solution. The dipper in which the vial sits is lowered to the bottom of the chamber

The effect of changing volume for this combination of dose calibrator and measurement vessel was examined using ionic ^90^Y in solution. A carrier free sample of ^90^Y chloride (Perkin Elmer, USA) with a calibrated radioactivity level of 4620 MBq in 1 ml was obtained. Assay of the sample was performed by the supplier without an uncertainty estimate and the only radioactive impurity reported was Sr-90 which compromised < 0.0001% of the sample at the time of calibration. The activity was transferred via a syringe to a 10 ml Wheaton vial and the activity was diluted by the addition of 5 g of sterile water for injection. Into each of the six Sirtex shipping vials was transferred 1 g of ^90^Y solution. Assay of the activity in the six vials indicated that less than 5% of the activity was lost in the transfer process. The vials were weighed using the balance mentioned previously and assayed using the CRC 55 TW calibrator by placing the vial manually in the centre of the “dipper” which was lowered into the dose calibrator as shown in Fig. 1. The dose calibrator chamber has a diameter of 6.1 cm and a depth of 25.4 cm, a plastic insert is used to protect the chamber from damage and contamination. The Sirtex shipping vial has a wall thickness of 2.0 mm and bottom thickness of 2.4 mm [13]. Water for injection was added to the vials in increments of 0.25 g up to 1 g total added and then in 1 g increments up to the final weight of 5 g with the vial being weighed and the radioactivity assayed in the dose calibrator at each weight. The dose calibrator measurements of radioactivity as a function of source weight were fitted with a “one phase” decay using non-linear regression (Graphpad Prism 6, Graphpad Inc). The one-phase decay function has the form.

$$y=\left( {y(0) - y(\infty )} \right){e^{ - kx}}+y(\infty )$$

where *y* is the reading of the dose calibrator as a function of the weight of solution in the vessel, *k* is an exponential decay constant and *x* is the weight of the solution. The fitted curves were normalized to the assay reading at 5 g to provide the vial-specific weight-response correction factors for measurements at weights of less than 5 g.

### Derivation of solution volume

Taking the density of the solution to be 1 g/ml, the dose calibrator response as a function of volume can be derived. It is recognized that to maintain a constant density, carrier added solution must be added to the solution instead of ordinary water however the lack of a carrier added solution will cause only negligible distortions of the solution density. The mass of yttrium chloride in the initial sample would have been less than 2 × 10^− 7^ g which is considered to have a negligible effect on the density of the solution in this situation. There is also a slight variation in water density based on impurities found in water for injection and changes in room temperature. These affects are considered negligible in comparison to the other affects considered in the uncertainty budget for the measurement of ^90^Y solution.

### Estimation of uncertainty in clinical practice

In order to gauge the significance of the response of the chamber an estimate of the uncertainty in clinical assay of ^90^Y solution was made. A standard reference material was obtained from ANSTO with a stated uncertainty of 1.8% which was directly traceable to the work reported by Mo et al. [14]. Other components of uncertainty were estimated for the CRC 55 TW calibrator used in this experiment.

### Assay of normalized activity in microsphere suspension

As we were unable to change the density of the resin microspheres directly, the combined effect of microsphere concentration and source volume was measured using a sample of SIR-Spheres (Sirtex Medical Limited, Sydney, Australia). The sample of SIR-Spheres was obtained with a calibrated activity of 1.8 ± 0.2 GBq as measured by the supplier. To estimate the uncertainty in the assay of the radioactive sample in our dose calibrator, the radioactivity in the vial was measured 10 times with the microspheres allowed 2 min to settle between measurements before being re-suspended and remeasured. Measurements were taken by placing the vial in the centre of the plastic “dipper” [7] immediately after agitation with the device being read out immediately before the spheres begin to settle. The microspheres were then allowed to settle to the bottom of the vial overnight. Using a 19G 90 mm spinal needle, 3.8 ± 0.1 g of water was slowly removed from the vial leaving the microspheres in approximately 1.2 g of water. The water removed from the vial was assayed in the dose calibrator to ensure that no ^90^Y was also removed in the process. The vial of microspheres was weighed before suspending the spheres and then measuring the activity in the CRC 55 TW. Water was added to the vial in approximately 0.25 g increments by syringe with the vial weighed, microspheres suspended and activity measured after each addition of water. The total weight of the suspension was increased to more than 5 g by the addition of the water. In this way the total activity in the vial remained constant but the concentration of microspheres varied fourfold. The volume of the suspension can be derived from the mass of added water as in the previous section and by using the manufacturer reported volume of 5 ml for the dose before the water was removed. The response of the chamber to varying quantity of water added to the microspheres was plotted alongside the response curve described previously.

## Results

### Weights

The empty vials had an average weight of 20.013 g (range 19.960–20.293 g). The difference between vials gave rise to a variation in ionization chamber response which is included in the uncertainty budget assuming a rectangular distribution.

### Volume correction curve

Five of the six vials that were previously prepared were used to calculate vial-specific radioactivity/volume response curves with one vial being excluded from the study when its walls became contaminated with radioisotope. There was little variation in the volume curves between vials with the greatest discrepancy being observed at small volumes (Table 1).

**Table 1:** Correction factors for volume effect for the five vials Sirtex shipping vials

Volume (ml)	Correction factor				
	Avg	Min	Max	Std dev	CoV (%)
1	1.136	1.134	1.137	0.001	0.13
2	1.057	1.055	1.058	0.001	0.11
3	1.021	1.021	1.023	0.001	0.08
4	1.007	1.006	1.007	0.0003	0.04
5	1	N/A	N/A	N/A	N/A

The correction factors were obtained by normalizing the dose calibrator reading to the reading at 5 ml. Correction factors were obtained for all five vials and the average (Avg), minimum (min), maximum (max), standard deviation (std dev) and coefficient of variation (CoV) are reported for the 1,2,3,4 and 5 ml volume

**Fig. 2:**
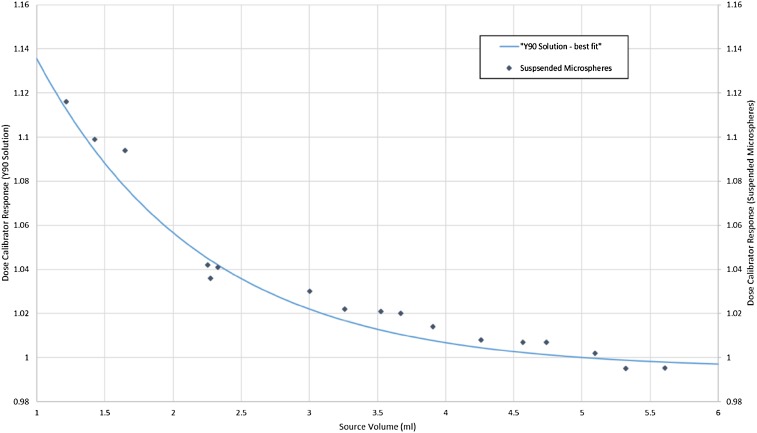
The reading of the dose calibrator normalized to a volume of 5 ml for both the suspended microspheres in a Sirtex shipping vial (data points) and the best fit of ^90^Y solution in a Sirtex shipping vial (blue curve) measured on a Capintec 55 TW. The response is the reading of the dose calibrator relative to the reading at a 5 ml source volume

The response of the dose calibrator (normalised to 5 ml) was given by

$$R\left( V \right)=\left( {1.315 - 0.99472} \right){e^{ - 0.8216V}}+0.99472$$

where *V* is the volume of the source. The correlation coefficient for the one-phase decay curve with the measured dose calibrator reading was greater than 0.99 for all five vials. The fitted curve is shown in Fig. 2. Fitting the data to a polynomial function was also explored but the correlation coefficient was poorer than the one-phase decay.

### Combined volume and concentration curve

Dose calibrator readings were plotted as a function of volume (Fig. 1) while keeping the number of microspheres fixed (i.e., varying concentration of microspheres) and the response of the dose calibrator was normalized to a volume of 5 ml. The data points from the dose calibrator readings differed from the best fit curve by less than 2%. This was within the uncertainty estimate for the assay of radioactive microspheres given in Table 2.

**Table 2:** The uncertainty budget for the clinical measurement of ^90^Ychloride solution in the Sirtex shipping vial

Source of uncertainty	Relative standard uncertainty (%)
Calibration factor	1.80
Dose calibrator resolution	0.28
Counting statistics	0.30
Background variability	0.04
Stability	0.58
Linearity	0.58
Variation between vials	2.3
Combined standard uncertainty (k = 1)	3.06

The coverage factor (k = 1) corresponds to a confidence level of 68%

## Discussion

The small variation in the characteristics of the five Sirtex shipping vials suggests that using a single volume adjustment curve for all vials of resin-based ^90^Y-labelled microspheres will make negligible difference to the shape of the volume correction curve. The variation in mass of the vials was consistent with other publications [13, 15] but in this instance the difference between maximum and minimum ionization chamber response was about 2% which is smaller than the 5–14% difference in chamber response reported by Thiam et al. [13] and less than the 3.4% uncertainty in variation between vials reported by Ferreira et al. [15]. Other studies have shown that different dose calibrators can have different sensitivity to the variation in vial thickness which can cause a 4% difference in chamber response [13]. The relative response of the Capintec 55 TW to vial and volume effects in ^90^Y solution and microspheres has not previously been reported in the literature and it may be the case that this model is less affected by these issues. The volume of the vial’s contents cannot be measured by weight because the density of the microspheres in suspension is unknown. No validated methods of measuring the volume of the vial contents are available so the uncertainty due to the volume cannot be overcome.

The volume curve is similar to that previously reported for 10 ml glass vials [12] and is similar to the change in chamber response to the source volume reported by Thiam et al. [13] who also used vials supplied by Sirtex. The asymptotic nature of the curve after approximately 5 ml in their study suggests that volume effects can be ignored if very small volumes are withdrawn or water is added to adjust the volume to 5 ml.

The good fit between the volume effect and the combined volume and concentration effect means that any error caused by the concentration of microspheres in the suspension will be negligible in the clinical context. It has been suggested that this can be attributed to the similar densities between the resin microspheres and water [14] with the density of resin microspheres reported as 1.06 g cm^− 3^ [13]. This fact suggests that an accurate assay of resin-based ^90^Y microspheres in the glass shipping vial can be obtained using a calibration factor derived from ^90^Y in solution. The estimate of uncertainty that we obtained for ^90^Y chloride solution is smaller than what was estimated by Ferreira et al. which is attributed to a smaller change in chamber response between vials [15]. Measurement of the residual activity in the vial can be performed accurately if the over-response of the dose calibrator at low volumes is corrected. Alternatively the vial can be refilled to 5 ml by the addition of water and the reduced concentration of microspheres will not have a significant effect on the dose calibrator reading.

It should be noted that Mo et al. [14], Lourenco et al. [16], Ferreira et al. [15] and Thiam et al. [13] all measured the activity of the microspheres in the settled state. This allows for a much larger number of measurements of the activity and hence a greater precision. In clinical practice this might not be used because it takes several minute for the spheres to settle [15] which can be difficult to accommodate in the clinical world and because the instructions from the manufacturer require measurement of the activity to be made with the spheres in the suspended state [17]. Measurements in the suspended state will therefore have less precision and so it is less likely that minor effects associated with the concentration of the spheres will be detected.

## Conclusion

Dose calibrators are sensitive to changes in the volume of the source when measuring ^90^Y-labelled resin microspheres, with a change in volume from 5 ml down to 1 ml leading to a 12% error in the reading. This paper has demonstrated a novel method for comparing the relative effect of density changes with variation of volume changed. In this example, using the case of resin based microspheres, the ionization chamber calibration factor is not dependent on microsphere density. An accurate assay of resin-based ^90^Y labelled microspheres is possible if the glass shipping vial is used as the calibration vessel and the volume effect is corrected, or the volume returned to the original 5 ml by adding water assuming the residual activity does not fall below the region of linear response of the dose calibrator.
